# Supplementary figures and images for: Dysfunction of Complementarity Determining Region 1 Encoded by T Cell Receptor Beta Variable Gene Is Potentially Associated with African Swine Fever Virus Infection in Pigs
Source: Microorganisms. 2024 May 30;12(6):1113. doi: 10.3390/microorganisms12061113 (PMC11205859; doi:10.3390/microorganisms12061113)

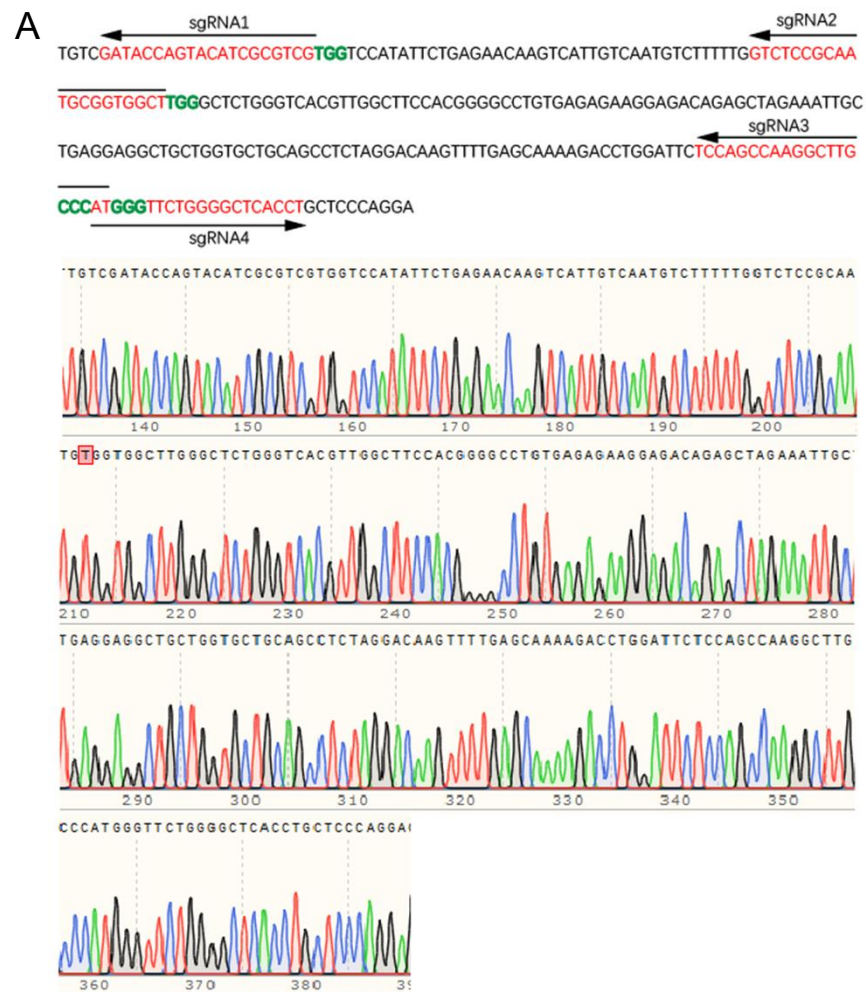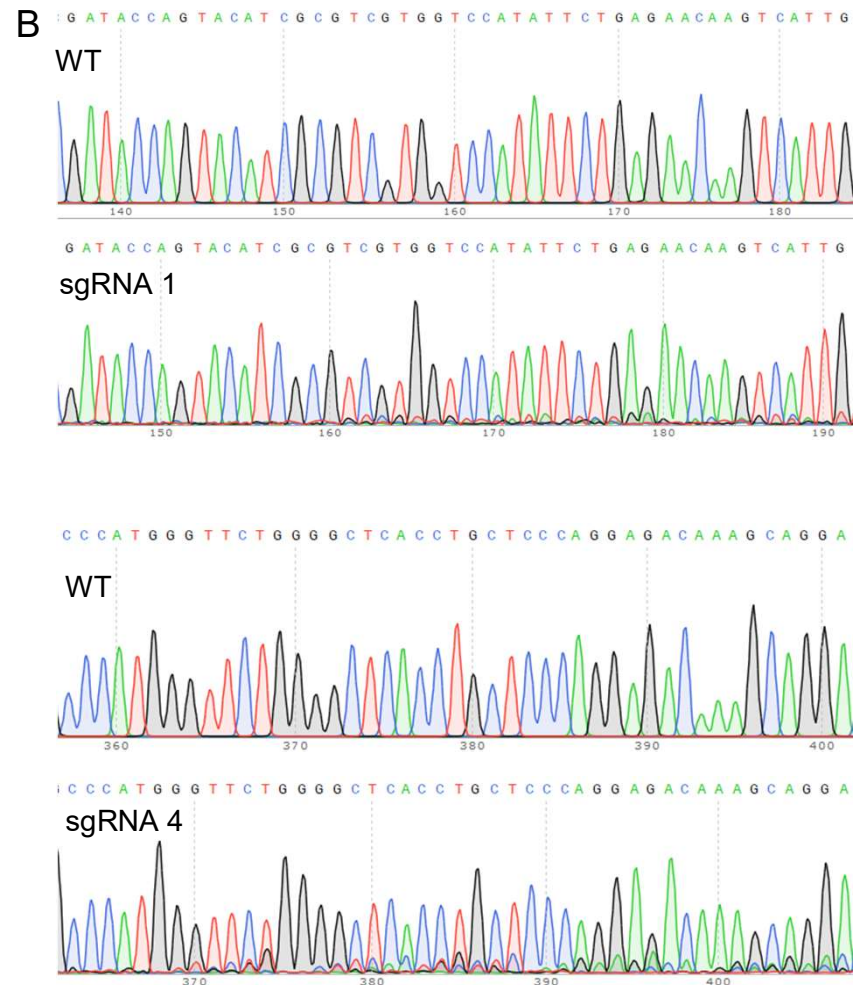

Supplement: Supplementary file 1 [file microorganisms-12-01113-s001.zip › microorganisms-2988728-Supplementary-05-15-2024/Microorgnism-2988728-FigureS1-05-22-2024.pdf]

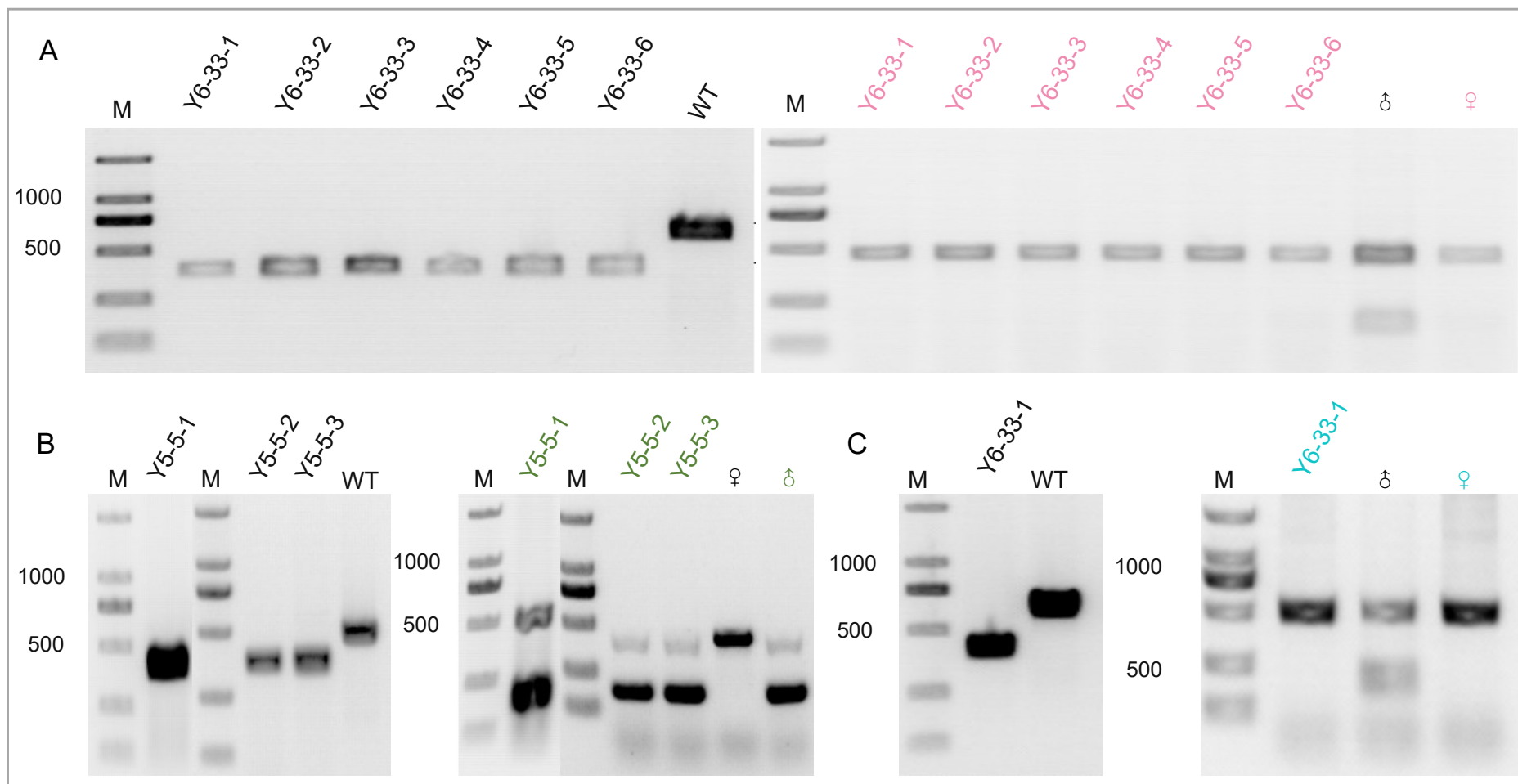

Supplement: Supplementary file 1 [file microorganisms-12-01113-s001.zip › microorganisms-2988728-Supplementary-05-15-2024/Microorgnism-2988728-FigureS2-05-22-2024.pdf]

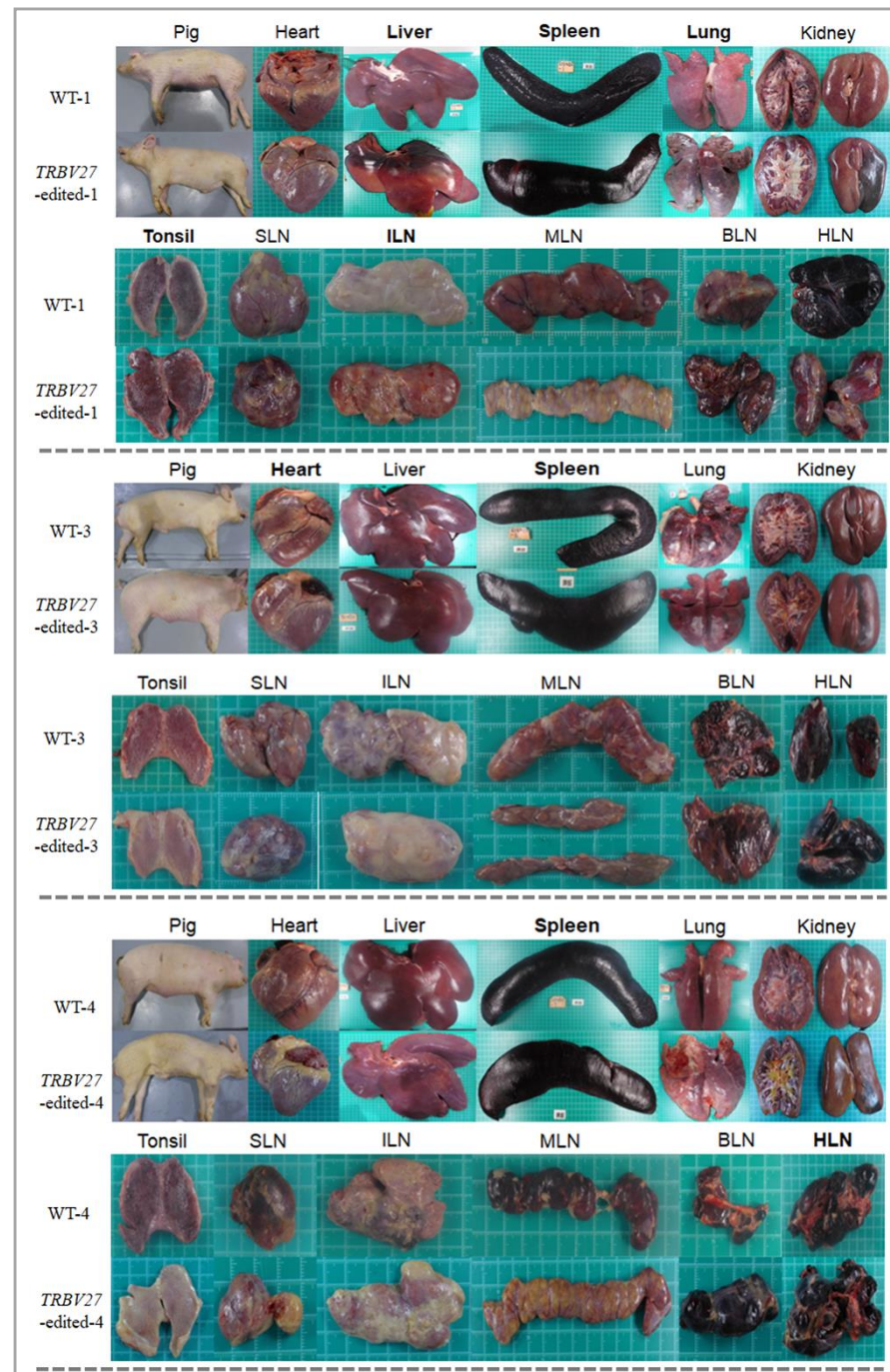

Supplement: Supplementary file 1 [file microorganisms-12-01113-s001.zip › microorganisms-2988728-Supplementary-05-15-2024/Microorgnism-2988728-FigureS3-05-22-2024.pdf]
